# Immunolocalization of serotonin in Onychophora argues against segmental ganglia being an ancestral feature of arthropods

**DOI:** 10.1186/1471-2148-7-118

**Published:** 2007-07-15

**Authors:** Georg Mayer, Steffen Harzsch

**Affiliations:** 1 Department of Anatomy and Cell Biology, University of Melbourne, Victoria 3010, Australia; 2 Department of Evolutionary Neuroethology, Max-Planck Institute for Chemical Ecology, Hans-Knöll-Straße 8, D-07745 Jena, Germany

## Abstract

**Background:**

Onychophora (velvet worms) represent the most basal arthropod group and play a pivotal role in the current discussion on the evolution of nervous systems and segmentation in arthropods. Although there is a wealth of information on the immunolocalization of serotonin (5-hydroxytryptamine, 5-HT) in various euarthropods, as yet no comparable localization data are available for Onychophora. In order to understand how the onychophoran nervous system compares to that of other arthropods, we studied the distribution of serotonin-like immunoreactive neurons and histological characteristics of ventral nerve cords in *Metaperipatus blainvillei *(Onychophora, Peripatopsidae) and *Epiperipatus biolleyi *(Onychophora, Peripatidae).

**Results:**

We demonstrate that paired leg nerves are the only segmental structures associated with the onychophoran nerve cord. Although the median commissures and peripheral nerves show a repeated pattern, their arrangement is independent from body segments characterized by the position of legs and associated structures. Moreover, the somata of serotonin-like immunoreactive neurons do not show any ordered arrangement in both species studied but are instead scattered throughout the entire length of each nerve cord. We observed neither a serially iterated nor a bilaterally symmetric pattern, which is in contrast to the strictly segmental arrangement of serotonergic neurons in other arthropods.

**Conclusion:**

Our histological findings and immunolocalization experiments highlight the medullary organization of the onychophoran nerve cord and argue against segmental ganglia of the typical euarthropodan type being an ancestral feature of Onychophora. These results contradict *a priori *assumptions of segmental ganglia being an ancestral feature of arthropods and, thus, weaken the traditional Articulata hypothesis, which proposes a sistergroup relationship of Annelida and Arthropoda.

## Background

**Figure 1:**
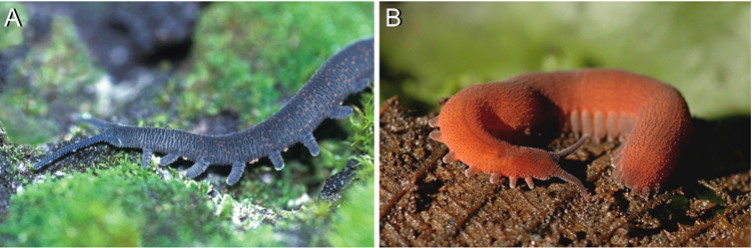
**Representatives of two major onychophoran taxa used for this study**. (A) Anterior end of a walking specimen of *Metaperipatus blainvillei *(Onychophora, Peripatopsidae) from Chile. (B) *Epiperipatus biolleyi *(Onychophora, Peripatidae) from Costa Rica.

Onychophora (or "velvet worms", Figure 1A and 1B) are commonly regarded as the sistergroup of Euarthropoda, i.e. Chelicerata, Myriapoda, Crustacea, and Hexapoda [1-6]. Morphologically onychophorans closely resemble the extinct Cambrian lobopodians, which is why they are by some authors considered as "living fossils" [7]. Due to their basal position within the Arthropoda, Onychophora represent a key group for the current discussion on the evolution of arthropods [8-11].

**Figure 2:**
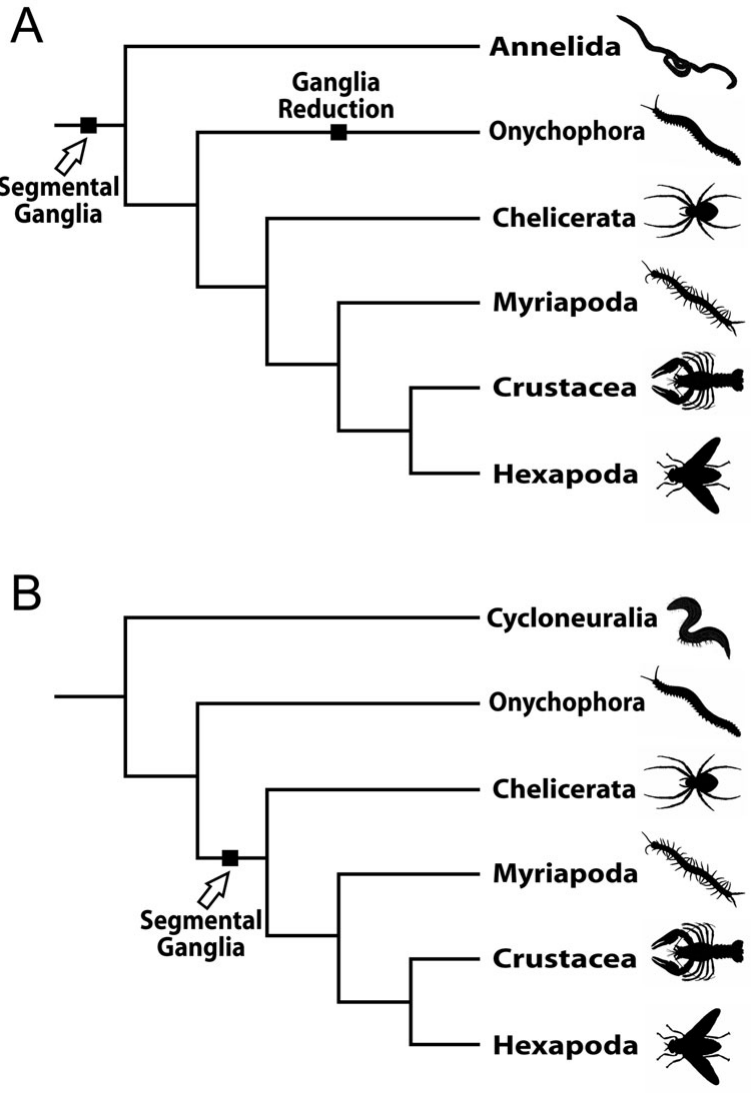
**Two competing hypotheses on phylogenetic position of arthropods and their bearing on the evolution of segmental ganglia**. (A) Articulata hypothesis: Arthropoda and Annelida are sistergroups. Segmental ganglia represent one of the synapomorphies uniting these taxa, but must have been reduced in Onychophora and modified in some annelids. (B) Ecdysozoa hypothesis: Arthropoda and Cycloneuralia (nematodes, priapulids, and allies) are sistergroups. Segmental ganglia evolved within the Arthropoda after the separation of Onychophora from the remaining arthropod groups.

Currently, there are two competing hypotheses concerning the phylogenetic position of Arthropoda within the Bilateria: (1) the traditional Articulata concept, and (2) the Ecdysozoa hypothesis [10,12-14]. In the controversial debates on the validity of either hypothesis, the structure of the nervous system is one of the most contentious issues [9,12,14-16]. According to the Articulata hypothesis (Figure 2A), which proposes a sistergroup relationship of Annelida and Arthropoda, a "rope ladder-like" nervous system (not to be confused with the "ladder-like" organization in flatworms and some other bilaterians), is a synapomorphy of these taxa [1,12,13,17-19]. The rope ladder-like nervous system is characterized by segmental, bilaterally arranged ganglia, which are longitudinally linked by connectives (axonal tracts without associated neuronal cell bodies) and transversely linked by commissures [20,21]. In many arthropods, the bilateral ganglia in each body segment have been fused, thus forming unpaired structures. Yet, according to the competing Ecdysozoa hypothesis (Figure 2B), segmental ganglia do not necessarily represent an ancestral feature of arthropods since they are absent in their next relatives, i.e. nematodes, priapulids, and allies. Only in kinorhynchs, which show an orthogonal nervous system, the mid-dorsal and mid-ventral longitudinal nerve cords are ganglionated [22]. Under the Ecdysozoa concept, segmental condensations of neuronal cell bodies, i.e. ganglia, might therefore have evolved convergently in Annelida and Arthropoda [21].

Notably, segmental ganglia, as seen in euarthropods, do not occur in Onychophora [23-25]. According to the Articulata hypothesis, segmental ganglia must thus have been reduced or modified in this taxon (Figure 2A; [12,18,19]). Based on studies of the nervous system in Onychophora, however, there are contrary views as to whether the structural correlates or "rudiments" of segmental ganglia occur in this group [24,25]. If no such correlates or remnants exist in Onychophora, this feature would not be useful for supporting the homology of segmentation in Annelida and Arthropoda [1,12,13,18], which would seriously compromise the Articulata hypothesis.

Immunolocalization of serotonin seems to be well suited to explore segmental characteristics of nerve cords in a phylogenetic context. In representatives of Echiura, which lack external body segmentation, recent immunocytochemical studies have shown that serotonin-like immunoreactive (5-HT-lir) neurons are nevertheless arranged in repeated units along the ventral nerve cord [26,27]. These repeated units were suggested to correspond to the typical segmental ganglia in Annelida, suggesting that the ventral nervous system was primarily segmented in Echiura and that this taxon might be closely related to or may even represent an in-group of Annelida [27]. This assumption is supported by the phylogenetic analyses of different molecular data sets [28-32]. Similar repeated sets of 5-HT-lir neurons occur in the ventral nervous system of the earthworm *Lumbricus terrestris *[33], an annelid species with secondarily modified segmental ganglia. Comparable evidence of repeated sets of individually identifiable 5-HT-lir neurons in Onychophora would suggest that segmental ganglia are an ancestral feature of Arthropoda (albeit modified in Onychophora), supporting the Articulata rather than the Ecdysozoa hypothesis. Onychophora, thus, represent an important group for understanding the evolution of nervous systems in arthropods.

The arrangement and architecture of serotonin-like immunoreactive neurons has been analyzed in a wide range of different arthropod species (reviews [34,35]). In all representatives of Euarthropoda studied thus far, segmentally iterated and bilaterally symmetric identifiable sets of 5-HT-lir neurons are present [34-38]. Although biogenic amines, including serotonin, have been shown to be present in the onychophoran nervous system by histochemical methods [39,40] and immunolocalization studies in the brain [41,42], no data are as yet available on the arrangement of 5-HT-lir neurons within the ventral nerve cord.

This study describes the general morphology of the ventral nerve cord and the distribution of 5-HT-lir neurons in two representatives of the major onychophoran taxa, Peripatidae and Peripatopsidae. We confirm that the onychophoran nerve cord is of medullary type characterized by a scattered distribution of neuronal cell bodies along the antero-posterior body axis and the absence of somata-free connectives. In addition, we do not find any serially iterated arrangement of 5-HT-lir neurons, suggesting that segmental ganglia are primarily absent and not secondarily reduced in Onychophora. This finding clearly weakens the traditional Articulata hypothesis as it shows that a chain of ventral ganglia most likely does not represent an ancestral feature of arthropods.

## Results

### General morphology of ventral nerve cords

**Figure 3:**
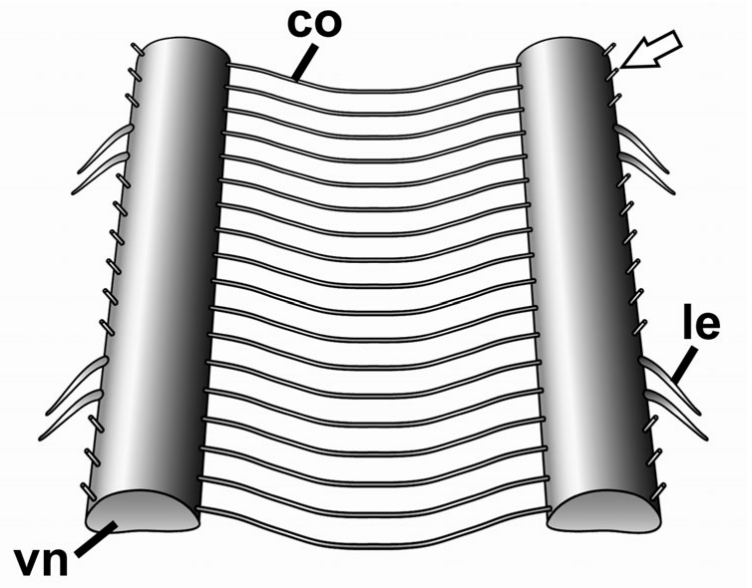
**Ventral nervous system in Onychophora**. Schematic illustration of ventral nerve cords (vn) and associated nerves within two segments of the trunk. Arrow points to one of the peripheral nerves innervating the body wall musculature (only nerve roots are shown). Abbreviations: co, commissure; le, leg nerve; vn, ventral nerve cord.

**Figure 4:**
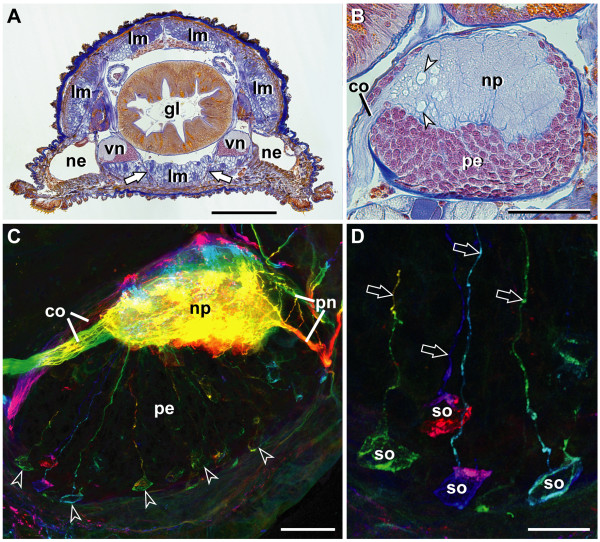
**Characteristics of ventral nerve cords and distribution of serotonin-like immunoreactive neurons in *Metaperipatus blainvillei *(Onychophora, Peripatopsidae)**. (A) Histological cross-section of a mid-body segment (Azan staining, adult specimen) to show the ventrolateral position of the nerve cords (vn) widely separated from each other. Arrows point to a median commissure connecting the nerve cords. Scale bar: 500 μm. (B) Histological cross-section of a nerve cord (Azan staining, adult specimen). Dorsal is up, median is left. Arrowheads indicate giant fibers within the neuropil (np). Scale bar: 100 μm. (C) Serotonin-like immunoreactivity within the nerve cord of an embryo almost ready for birth (vibratome section). Dorsal is up, median is left. Neuropil contains a high number of 5-HT-lir fibers. Most somata of 5-HT-lir neurons (arrowheads) are situated in the periphery of the perikaryal layer (pe). Depth-coded image of stacks of confocal micrographs (75 × 0.75 μm). The colors range from warm (red/yellow) indicating anterior optical sections to cooler (blue/purple) indicating posterior optical sections. Scale bar: 50 μm. (D) Details of 5-HT-lir neurons within a nerve cord of the same specimen (as in C). Each neuron gives rise to a single neurite that targets the neuropil without fasciculating with other fibers (arrows). Scale bar: 20 μm. Abbreviations: co, commissure; gl, gut lumen; lm, longitudinal musculature; ne, nephridial end bladder; np, neuropil; pe, perikaryal region; pn, peripheral nerves; so, somata of 5-HT-lir neurons; vn, ventral nerve cords.

**Figure 5:**
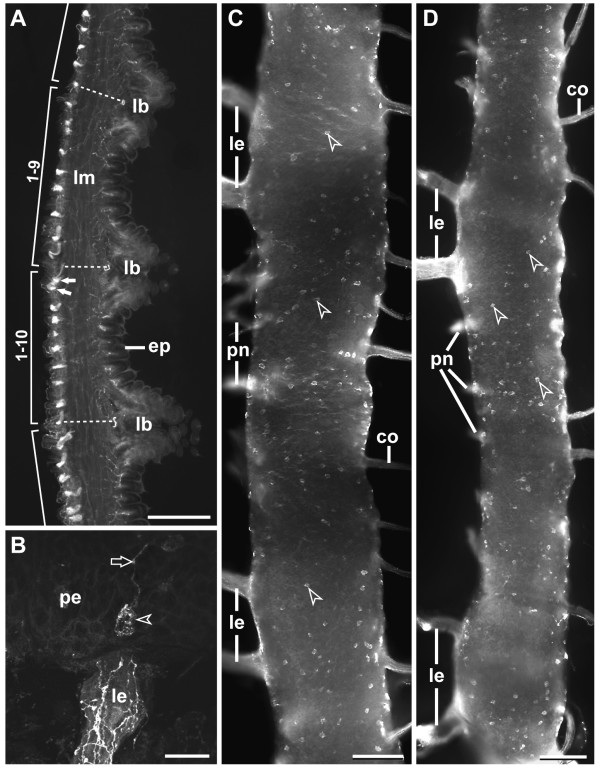
**Serotonin-like immunoreactivity in median commissures and ventral nerve cords of *Metaperipatus blainvillei *(Onychophora, Peripatopsidae), micrographs from adult specimens**. (A) Fluorescent micrograph of a sagittal vertical vibratome section of the ventrolateral body wall in three leg-bearing segments. Dorsal is to the left, ventral to the right. Dotted lines indicate the dorso-ventral axes of legs, which are sectioned near their bases (lb). The median commissures are arranged regularly throughout the onychophoran body. Nine and ten commissures are seen in two subsequent segments, respectively. Note that two of the commissures are close to each other (small white arrows). Scale bar: 300 μm. (B) Detail of a 5-HT-lir cell body (arrowhead) and its single projection (arrow) situated close to a leg nerve (le). Confocal laser scanning micrograph. Scale bar: 20 μm. (C, D) Whole-mount preparations of ventral nerve cords in ventral view. Two subsequent segments are illustrated in each fluorescent micrograph. Peripheral nerves and several median commissures have been obliterated during preparation. Median is to the right, lateral to the left. The somata of 5-HT-lir neurons (arrowheads) are scattered throughout the nerve cords and do not show any metameric arrangement. Scale bars: 100 μm. (C) Leg-bearing segments 11–12. (D) Leg-bearing segments 17–18. Abbreviations: co, commissures; ep, epidermis; lb, leg basis; le, leg nerves; lm, longitudinal musculature; pe, perikaryal layer; pn, peripheral nerves.

**Figure 6:**
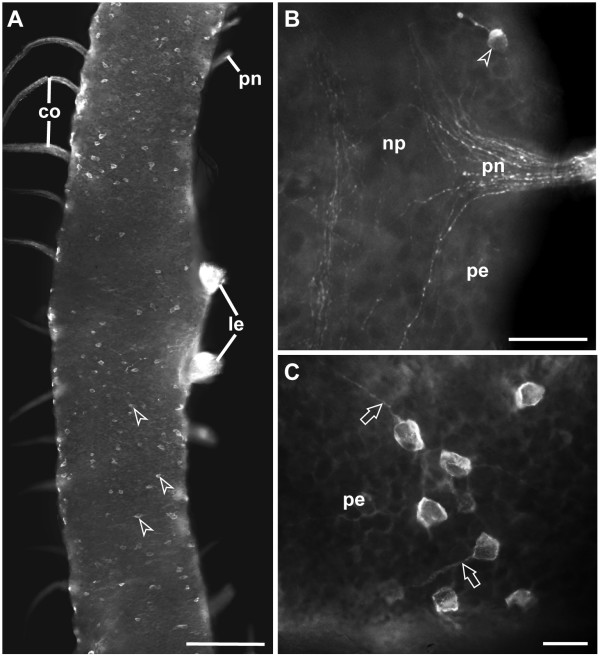
**Serotonin-like immunoreactivity in ventral nerve cords of *Epiperipatus biolleyi *(Onychophora, Peripatidae), fluorescent micrographs from adult specimens**. (A) Whole-mount preparation of a ventral nerve cord in ventral view. Median is to the left, lateral to the right. Distal portions of leg nerves and peripheral nerves have been obliterated during preparation. The somata of 5-HT-lir neurons (arrowheads) do not form any clusters or an identifiable pattern but are instead irregularly scattered along the nerve cord. Scale bar: 200 μm. (B) Detail of a whole-mount preparation of a nerve cord in dorsal view. 5-HT-lir fibers leave the neuropil (np) *via *a peripheral nerve (pn). Arrowhead indicates a unipolar 5-HT-lir neuron. Scale bar: 50 μm. (C) Detail of peripherally situated somata of unipolar 5-HT-lir neurons (ventral view). Like in *Metaperipatus blainvillei*, the neurites of 5-HT-lir neurons (arrows) do not fasciculate with other fibers in *E. biolleyi *but run as single processes towards the neuropil. Scale bar: 20 μm. Abbreviations: co, commissures; le, leg nerves; np, neuropil; pe, perikaryal layer; pn, peripheral nerves.

The bilaterally paired ventral nerve cords of onychophorans represent longitudinal structures that are connected with each other by numerous commissures (see schematic in Figure 3 and Figures 4A and 4B). Typically, there are nine commissures per body segment, but their number varies from eight to ten in different segments (see, e.g., Figure 5A, further data not shown). Herein, each segment is characterized by the position of legs and associated structures (e.g. leg nerves), as there are no distinct metameric landmarks in the inter-pedal regions of the onychophoran body. At regular intervals, each cord gives rise to a pair of lateral nerves innervating the legs (Figure 3; Figures 5C and 5D; Figure 6A). Additional peripheral nerves supplying the body wall musculature originate in the inter-pedal regions of successive segments (Figure 3; Figure 4C; Figures 5C and 5D; Figures 6A and 6B). Within the body, the nerve cords flank the gut in a ventrolateral position (Figure 4A). They are dorsoventrally flattened and appear oval in shape in cross-sections (Figure 4B). The internal structure of each nerve cord is uniform throughout the body, comprising a dorsal neuropil and a ventral, ventromedian and ventrolateral layer with neuronal somata (Figures 4A and 4B). The median part of the neuropil bears numerous giant fibers, two of which are larger than the others (arrowheads in Figure 4B; see also [43]). On its outer surface, i.e. towards the body cavity, each nerve cord is enveloped by a thick layer of collagenous connective tissue.

Observations on several specimens of nerve cords of *Metaperipatus blainvillei *and *Epiperipatus biolleyi *sectioned in sagittal, horizontal, and transverse planes, or examined as whole-mount preparations (*E. biolleyi*: 3 specimens; *M. blainvillei*: 22 specimens) failed to reveal distinct segmental thickenings (cf. Figures 5C and 5D; Figure 6; further data not shown).

### Serotonin-like immunoreactivity

In accordance with earlier physiological and biochemical studies [39,40] our data show that 5-HT-lir material is widely distributed throughout the ventral nerve cords in both species studied: *Metaperipatus blainvillei *and *Epiperipatus biolleyi *(Figures 4C and 4D; Figures 5A, 5B, 5C, and 5D; Figures 6A, 6B and 6C). No species-specific differences in the distribution patterns were observed. The neuropil contains a dense network of 5-HT-lir fibers (Figure 4C). Labeled fibers exit the neuropil *via *the median commissures towards the contralateral cord, *via *the paired lateral leg nerves, and *via *peripheral nerves innervating the body wall (Figure 4C; Figures 5A, 5B, 5C and 5D; Figures 6A and 6B). 5-HT-lir neurons were exclusively unipolar (Figures 4C and 4D; Figure 5B; Figures 6B and 6C). Within the perikaryal layer of each nerve cord, the somata of 5-HT-lir neurons are arranged mainly in the periphery, i.e., near the ventral and ventrolateral surface of the nerve cords (Figures 4C and 4D; Figures 5C and 5D; Figures 6B and 6C). The somata are small, measuring between 15–20 μm in size. Their single neurites project towards the neuropil without fasciculating with other processes (Figures 4C and 4D; Figure 5B; Figure 6C). The course of the neurites could not be followed within the neuropil because of the high level of labeling of 5-HT-lir fibers (cf. Figure 4C).

The analysis of vibratome sections as well as whole-mount preparations showed that in both onychophoran species studied the distribution of 5-HT-lir neurons does not exhibit any ordered arrangement (Figures 4C and 4D; Figures 5C and 5D; Figures 6A and 6C). The immunoreactive neurons (80–100 per hemisegment) are instead scattered randomly throughout the entire length of each nerve cord (Figures 5C and 5D; Figure 6A). Specifically, we neither observed segmentally iterated nor bilaterally symmetric patterns. In addition, we did not find any individually identifiable 5-HT-lir neurons.

## Discussion

This study revealed that in Onychophora, the arrangement of 5-HT-lir neurons does not show any recognizable serial or segmental organization, which is in contrast to the strict segmentally iterated arrangement of 5-HT-lir neurons in most other arthropods, including Chelicerata, Myriapoda, Crustacea, and Hexapoda (see reviews [34,35]). 5-HT-lir neurons are instead scattered throughout the entire length of each onychophoran nerve cord in an apparently random fashion. Serotonin-like immunoreactivity accordingly does not provide any evidence for modified ganglionic concentrations of neurons or even ganglion rudiments in Onychophora. Our data clearly support previous reports [23-25] in showing that the onychophoran nerve cord belongs to the medullary type ("Markstrang") of nervous systems that are characterized by a scattered distribution of neuronal cell bodies along the nerve cords and the absence of somata-free connectives (e.g. [20]). In this respect, the organization of the onychophoran nerve cord resembles that of the longitudinal nerve cords in other bilaterians, such as Platyhelminthes [44,45], Gnathostomulida [46], Nemertea [47], Mollusca [48], Nematoda [47], and some representatives of Annelida [20,21], rather than that in Euarthropoda. In Euarthropoda, typical bilaterally arranged segmental ganglia are present, which are longitudinally connected by connectives (axonal tracts without associated neuronal cell bodies) and transversely linked by commissures. This fact contradicts recent suggestions of segmental ganglia being a common or an ancestral feature of Onychophora (figure 7 in [49]; figure 3 in [50] and [16]; figure 14 in [41]). Furthermore, our data argue against the claims [16,50] that only two commissures per segment are characteristic of the onychophoran nervous system. Yet, the onychophoran medullary type nervous system, in which few elements e.g. the leg nerves (see below) are serially iterated, may have served as the starting point, from which the euarthropodan rope ladder-like nervous system with its typical segmental characteristics has evolved.

In contrast to previous neuroanatomical studies [24,51], we found that paired nerves innervating the legs represent the only segmentally arranged structures associated with the onychophoran nerve cord. This layout closely corresponds with the organization of musculature in Onychophora since muscles supplying the legs are the only segmental structures within the onychophoran muscular system [8,52,53]. The metameric thickenings described by Federow [24] correlate with the position of legs, which are partially hollow and provide additional space for the nerve cords to evade laterally when the animal's body is contracted. Although the commissures and peripheral nerves do show a repeated pattern, this organization is independent from the segmental arrangement of other structures and organs, i.e. legs, nephridia and heart ostia, which are typical for each onychophoran body segment [8,10,54]. In addition to the neuroanatomical data obtained from adult specimens, ganglia as discrete gross morphological entities are not apparent in the development of the onychophoran nerve cord [3,9,10,55-57].

## Conclusion

Taken together, studies on neuroanatomy and neurogenesis [9,55,57] in Onychophora do not provide any evidence that segmental ganglia or a rope ladder-like organization is an ancestral feature of the onychophoran nerve cord. Rather, this structure shows many similarities with the medullary or "Markstrang" type of many other representatives of Bilateria [21,47,48], including platyhelminths [44,45]. Whether this type of organization represents an ancestral or derived feature of Onychophora must be clarified by further comparative studies using immunolabeling methods with other neuron-specific markers. However, recent neuroanatomical studies on representatives of Annelida [21] also contradict previous assumptions that their nervous system originally had a rope ladder-like organization like that seen in Euarthropoda. This strongly suggests that a rope-ladder type of nervous system, comprising segmental ganglionic condensations linked by somata-free connectives, has evolved convergently in annelids and arthropods. Thus, our results as well as recent immunocytochemical findings on annelids are in stark contrast to previous views [1,12,17-19] that *a priori *suggest segmental ganglia to be one of the synapomorphies uniting the Annelida and Arthropoda. Therefore, the character "presence of segmental ganglia" can no longer be taken as supporting the monophyly of Articulata since these structures were most likely absent in the last common ancestor of Onychophora and Euarthropoda.

## Methods

### Animals

Specimens of *Metaperipatus blainvillei *(Gervais, 1837), a representative of the Peripatopsidae (Figure 1A), were obtained from rotten logs in a forest near Lago Tinquilco (Chile) in July 2004. Specimens of *Epiperipatus biolleyi *(Bouvier, 1902), a member of the neotropical Peripatidae (Figure 1B), were collected under moss and among plant roots growing along the sides of a dirt road in the area of Cascajal de Coronado, near San José (Costa Rica), in October 2005. The animals were kept in cultures before they were studied.

### Histology and light microscopy

For histological studies, specimens of *M. blainvillei *were anaesthetized in ethyl acetate vapor for a few minutes, fixed in Bouin's fluid for several weeks, dehydrated in an ethanol series, methylbenzoate and butanol, and embedded in paraplast (Kendall). Complete series of 5 to 7 μm thin sections were made with steel blades on a microtome (Reichert-Jung, 2050-supercut). The sections were stained with an Azan staining method [58] and analyzed with a light microscope (Olympus BX51) equipped with a color digital camera (Colour View II, SIS).

### Immunocytochemistry, fluorescence microscopy and confocal laser scanning microscopy

For immunocytochemistry, either dissected nerve cords or vibratome sections of the specimens were processed. For vibratome sections, tissue from the trunk of late embryos (shortly before birth), juveniles, and adult specimens of *M. blainvillei *were fixed overnight in 4% paraformaldehyde (in 0.1 M phosphate-buffered saline, pH 7.4) at room temperature. The tissue was then rinsed in several changes of 0.1 M phosphate-buffered saline (PBS) containing 0.05% sodium azide and kept therein for several days. After removing the gut and slime glands, the tissue containing nerve cords was embedded in 7% agarose gel at 60°C and cooled to room temperature. The trimmed blocks were sectioned into series of 60–80 μm thin sections with steel blades on a vibratome (V 1000 plus Sectioning System, Vibratome Company). Sections were washed in several changes of PBS and then pre-incubated in PBS-TX (1% bovine serum albumin, 0.05% sodium azide, and 0.5% Triton X-100 in PBS) for 2 h at room temperature. Incubations with the primary antibody (rabbit anti-serotonin, ImmunoStar Incorporated; diluted 1:1,000 in PBS-TX) were carried out overnight at room temperature. The specimens were then washed in several changes of PBS and incubated overnight in a secondary antibody (anti-rabbit, conjugated to Alexa 488, Molecular Probes; diluted 1:500 in PBS-TX). After washing in PBS, the sections were mounted on glass slides in Gel Mount (Sigma).

For whole-mount immunocytochemistry, dissected nerve cords of *M. blainvillei *and *E. biolleyi *were dehydrated through an ethanol series (50%, 70%, 95%, 2 × 100%; 6 min each) and the sheaths of connective tissue enclosing each nerve cord made more permeable by incubation in xylene for 2 min. After re-hydration and several washes in PBS, the tissue was incubated in a NaBH_4 _solution (0.5% in PBS; 2 × 1 h) in order to minimize autofluorescence. All of the following steps were carried out at 37°C. After several washes in PBS, the sheath enclosing each nerve cord was digested with collagenase and hyaluronidase (Sigma; 1 mg/ml each in PBS + 0.5% Triton X-100) for 1 h. After washing in PBS, the tissue was pre-incubated in a blocking solution (10% bovine serum albumin + 0.5% Triton X-100 in PBS) for 2 h, and incubated with the primary antibody (rabbit anti-serotonin, ImmunoStar Incorporated; diluted 1:1,000 in PBS-TX) for 24 h. The incubation in the secondary antibody and mounting was carried out as described for vibratome sections.

Both the vibratome sections and whole-mounts were viewed with either a fluorescence microscope (Olympus BX61 equipped with an F-View II digital camera, SIS) or a confocal laser-scanning microscope (Zeiss LSM 410). Digital fluorescent images were processed with the LUCIA 4.82 and AnalySIS software packages, and Adobe Photoshop CS. The confocal images in this study are based on stacks of 75 optical sections of z-series taken at 0.75 μm intervals. Depth coded images were produced using ImageJ 1.34q.

### Specificity of antiserum

The antiserum against serotonin (ImmunoStar Incorporated, ; Cat. No. 20080, Lot No. 541016) is a polyclonal rabbit antiserum raised against serotonin coupled to bovine serum albumin (BSA) with paraformaldehyde. The antiserum was quality control tested by the manufacturer using standard immunohistochemical methods. According to the manufacturer, staining with the antiserum was completely eliminated by pretreatment of the diluted antibody with 25 μg of serotonin coupled to BSA. We performed an additional control and preadsorbed the diluted antiserum with 10 mg/ml BSA for 4 h at room temperature. This preadsorption did not affect the staining, thus, providing evidence that the antiserum does not recognize the carrier molecule alone. The anti-serotonin antiserum (formerly supplied by Immunonuclear Corp.) has thoroughly been tested in preadsorption controls by Beltz and Kravitz [59]. These authors preadsorbed the antiserum with the original serotonin-BSA conjugate that was used for generation of the antiserum, as supplied by the manufacturer. Preadsorption of the antibody in working dilution with formaldehyde cross-linked serotonin-BSA, in which the BSA concentration was 300 μg/ml, with a BSA:serotonin ratio of ca. 10:1 at 4°C for 24 h completely eliminated all staining [59]. The manufacturer also examined the cross reactivity of the antiserum. According to the data sheet, with 5 μg, 10 μg, and 25 μg amounts, the following substances did not react with the antiserum diluted to 1:20,000 using the horse radish peroxidase (HRP) labeling method: 5-hydroxytryptophan, 5-hydroxyindole-3-acetic acid, and dopamine. In control experiments for possible nonspecific binding of the secondary antiserum, we omitted the primary antiserum, replaced it with blocking solution, and followed the labeling protocol as above. In these cases, staining was absent. Despite this set of controls, we cannot fully exclude that the antiserum could be binding to serotonin-related substances in addition to serotonin. We therefore refer to the labeling that we observed as "5-HT-like immunoreactivity" or "5-HT-lir" throughout the text.

### Determination of neuron numbers

A precise number of 5-HT-lir neurons or statistics on their distribution cannot be given because of the ambiguity of segmental boundaries within the nerve cords. Instead, we provide an approximate range of neuron numbers per hemisegment (see "Serotonin-like Immunoreactivity" in the Results). This range is based on counts from 4 nerve cords with 20 segments in two specimens of *E. biolleyi *and 2 nerve cords with 15 segments in a specimen of *M. blainvillei*, respectively (see also Figures 5C and 5D; Figure 6A, in which the neuron numbers fell into the range given in the Results).

## Competing interests

The author(s) declare that they have no competing interests.

## Authors' contributions

GM obtained the animals and drafted the manuscript. Both authors carried out the immunocytochemical experiments and microscopic analyses, and approved the final manuscript.
